# Human adipose-derived stromal cells transplantation prolongs reproductive lifespan on mouse models of mild and severe premature ovarian insufficiency

**DOI:** 10.1186/s13287-021-02590-5

**Published:** 2021-10-10

**Authors:** Giulia Salvatore, Massimo De Felici, Susanna Dolci, Cosimo Tudisco, Rosella Cicconi, Luisa Campagnolo, Antonella Camaioni, Francesca Gioia Klinger

**Affiliations:** 1 Department of Biomedicine and Prevention, Section of Histology and Embryology, University of Rome Tor Vergata, Rome, Italy; 2 Fondazione Santa Lucia, IRCCS, Rome, Italy; 3 Department of Biomedicine and Prevention, Section of Human Anatomy, University of Rome Tor Vergata, Rome, Italy; 4 Department of Clinical Surgery and Translational Medicine, Sports Traumatology Unit, University Hospital of Rome Tor Vergata, Rome, Italy; 5 CIMETA, University of Rome Tor Vergata, Rome, Italy

**Keywords:** Adipose-derived stromal cells, POI, Chemotherapy, Fertility, Follicles, Stem cell transplantation

## Abstract

**Background:**

Although recent studies have investigated the ability of Mesenchymal Stromal Cells (MSCs) to alleviate short-term ovarian damage in animal models of chemotherapy-induced Premature Ovarian Insufficiency (POI), no data are available on reproductive lifespan recovery, especially in a severe POI condition. For this reason, we investigated the potential of MSCs isolated from human adipose tissue (hASCs), since they are easy to harvest and abundant, in ameliorating the length and performance of reproductive life in both mild and severe chemotherapy-induced murine POI models.

**Methods:**

Mild and severe POI models were established by intraperitoneally administering a light (12 mg/kg busulfan + 120 mg/kg cyclophosphamide) or heavy (30 mg/kg busulfan + 120 mg/kg cyclophosphamide) dose of chemotherapy, respectively, in CD1 mice. In both cases, a week later, 1 × 10^6^ hASCs were transplanted systemically through the tail vein. After four additional weeks, some females were sacrificed to collect ovaries for morphological evaluation. H&E staining was performed to assess stroma alteration and to count follicle numbers; immunofluorescence staining for αSMA was used to analyse vascularization. Of the remaining females, some were mated after superovulation to collect 2-cell embryos in order to evaluate their pre-implantation developmental capacity in vitro, while others were naturally mated to monitor litters and reproductive lifespan length. F1 litters’ weight, ovaries and reproductive lifespan were also analysed.

**Results:**

hASC transplantation alleviated ovarian weight loss and size decrease and reduced alterations on ovarian stroma and vasculature, concurrently preventing the progressive follicle stockpile depletion caused by chemotherapy. These effects were associated with the preservation of the oocyte competence to develop into blastocyst in vitro and, more interestingly, with a significant decrease of chemotherapy-induced POI features, like shortness of reproductive lifespan, reduced number of litters and longer time to plug (the latter only presented in the severe POI model).

**Conclusion:**

Human ASC transplantation was able to significantly reduce all the alterations induced by the chemotherapeutic treatment, while improving oocyte quality and prolonging reproductive functions, thus counteracting infertility. These results, strengthened by the use of an outbred model, support the potential applications of hASCs in women with POI, nowadays mainly induced by anticancer therapies.

**Graphic abstract:**

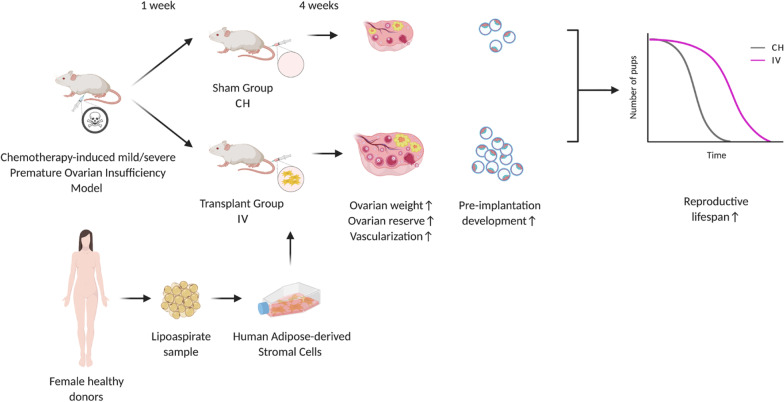

**Supplementary Information:**

The online version contains supplementary material available at 10.1186/s13287-021-02590-5.

## Background

It is widely accepted that mammalian females are born with a finite number of oocytes singularly enclosed within primordial follicles. These constitute the so-called ovarian reserve destined to progressively decrease until women are left with only about a thousand primordial follicles, which determines a woman’s entrance into menopause at approximately 50 years of age [1–3].

The onset of menopause before the age of 40 is defined as Premature Ovarian Insufficiency (POI). Such condition affects 1% of women under the age of 40, and 0.1% of women under the age of 30 [4]. Despite research carried out over decades to identify the cause of POI, more than half of the cases remain idiopathic. Around 20–25% of cases have a genetic cause, out of which approximately 9% of cases are due to aberrations in the X chromosome [5, 6]. Turner syndrome is the most common chromosomal aberration abnormality in patients with POI. Amongst the other genetic causes, fragile X premutation is the most well studied and occurs due to change in the *FMR1* gene (Xq27.3). One of the major non-genetic causes of POI is the exposure to iatrogenic agents, like chemotherapeutic treatment [7–10]. Alkylating agents such as busulfan and cyclophosphamide, are known to cause a marked depletion of the ovarian reserve, thus leading to premature infertility. The degree of depletion, and therefore the onset of POI, is dependent on several variables like individual susceptibility, patient age at time of treatment, dose and type of chemotherapeutic agent used [11]. With the improvement of early diagnosis and cancer treatment efficacy, five-year net survival for women experiencing cancer has greatly increased in most countries between 1995 and 2014 (approaching 90% in North America and Oceania). This is true especially for younger patients [12]. Women with POI clinically present with amenorrhea, hypergonadotropism and hypoestrogenism, other than infertility. As a consequence, POI is correlated with diverse health risks including osteoporosis, autoimmune disorders, ischemic heart disease, psychological distress and increased risk of mortality [8]. It is clear that quality of life after cancer treatment of this increasing number of women is becoming a very important issue to deal with. As a consequence, multiple lines of research are exploiting new and more efficient ways to overcome this problem.

Currently the only treatment approved for fertility preservation of oncologic patients is oocyte or embryo cryopreservation, which are techniques that arise several problems since they are expensive, ethically questionable, time-consuming, and not feasible for pre-pubertal girls or patients with hormone responsive cancers. In such cases the recommended option is cryopreservation of the ovarian cortex, although it is a technique that, like the administration of fertoprotective agents (e.g. GnRH-a), is still considered experimental in some countries and not completely safe [11, 13–17].

To overcome these problems, in recent years, stem cell transplantation has been investigated as a possible alternative to current POI treatments. Many preclinical studies were carried on to understand if Mesenchymal Stromal Cells (MSCs) derived from various tissues (i.e. bone marrow, adipose tissue, endometrium, amniotic fluid, amnion and chorion), could restore ovarian function in models of chemotherapy-induced POI [18]. A recent meta-analysis showed MSC transplantation can statistically increase ovarian weight, follicle count, pregnancy rate and E2 blood levels while lowering those of FSH [19]. Other studies further showed MSCs may decrease granulosa cell/stroma apoptosis [20–23], improve vascularization [21] and increase expression of AMH, FSHR and Ki67 [21, 24–26].

To our knowledge no studies actually analyzed the whole reproductive lifespan together with the offspring health and survival from MSC transplanted mothers; the analyses previously performed were limited to the study of implantation rate or the first litters delivered at the most [27–33]. Amongst these, in only two papers Adipose-derived Stromal Cells (ASCs), from rat, were used [29, 31]. In human, ASCs present a number of advantages, including abundance, ease of harvesting and minimally invasive recovering that facilitate autologous utilization over the most commonly used bone marrow MSCs [34].

In light of this, and given the diversity of protocols used to induce POI in mice [35, 36], we tested human ASC (hASCs) efficacy on reproductive performance in two murine models of both mild and severe chemotherapy-induced POI. Moreover, in order to produce results that more closely reflected the heterogeneity of human population, we chose outbred CD1 mice to set our model. Finally, given the evidence that some chemotherapeutic agents may have transgenerational effects [37], we also present initial results on F1 pups fertility.

## Methods

### hASC isolation and culture

hASCs were obtained slightly modifying a previously published protocol [38]. Briefly, lipoaspirate samples were collected from adult healthy women, after signed informed consent. Samples were washed in PBS and then digested overnight at 37 °C with 0.5 mg/ml Collagenase IA (Sigma-Aldrich). The Stromal Vascular Fraction (SVF) resulting from the centrifugation of the digested sample was then cultured in αMEM (Aurogene) supplemented with 10% FBS (Gibco), 2 mM L-glutamine, 100 UI/ml penicillin and 0.1 mg/ml streptomycin (all from Sigma-Aldrich) at 37 °C and 5% CO_2_ for 3–4 days. After this period, culture medium was changed every 2–3 days. When cells reached approximately 80% confluency they were detached by incubation with Trypsin–EDTA (Sigma-Aldrich) for 5 min and plated at a confluence of 1000 cells/cm^2^. All the procedures involving hASCs were performed under the authorization number 160/20 from the Ethical Committee of Fondazione PTV Policlinico Tor Vergata. In all experiments, cells were used at passage 3–5.

### hASC trilineage differentiation

For adipogenic differentiation, cells were plated at 30.000 cells/cm^2^ in DMEM supplemented with 10% FBS (Gibco), 2 mM L-glutamine, 0.5 mM IBMX, 50 μM indomethacin and 0.5 μM dexamethasone (all from Sigma-Aldrich) [39]. For osteogenic differentiation, cells were plated at 3.000 cells/cm^2^ in DMEM supplemented with 10% FBS (Gibco), 2 mM L-glutamine, 0.1 μM dexamethasone, 10 mM β-glycerophosphate and 50 μM ascorbate-2-phosphate (all from Sigma- Aldrich) [40]. For chondrogenic differentiation, 1 × 10^5^ cells were plated, using the micromass culture technique, in DMEM supplemented with 10% FBS (Gibco), 2 mM L-glutamine, 6.25 μg/ml human insulin, 10 ng/ml TGF-β1 (Peprotech), 50 nM ascorbate-2-phosphate (all from Sigma- Aldrich) [41]. Media was changed every 3–4 days for 21 days. Lipid accumulation was then confirmed through 0.2% Oil Red-O staining (Sigma-Aldrich). Matrix mineralization was confirmed through 40 mM Alizarin Red S staining (Sigma-Aldrich). Chondrogenic differentiation was confirmed, after sectioning the micromass, through 1% Alcian Blue 8GX staining of sulphated proteoglycan-rich matrix and cartilage lacunae identification.

### Cytofluorimetric analysis

For cytofluorimetric analysis 5 × 10^5^ cells were incubated with conjugated monoclonal anti-CD34, anti-CD45, anti-CD73, anti-CD90 and anti-CD105 (from MACS Miltenyi Biotec). Cells were analysed on a FACSCanto flow cytometer (BD Biosciences). 7-AAD was used to exclude non-viable cells from the analysis. Data were analysed using FACSDiva Software (BD Biosciences).

### Animals and treatments

**Fig. 1:**
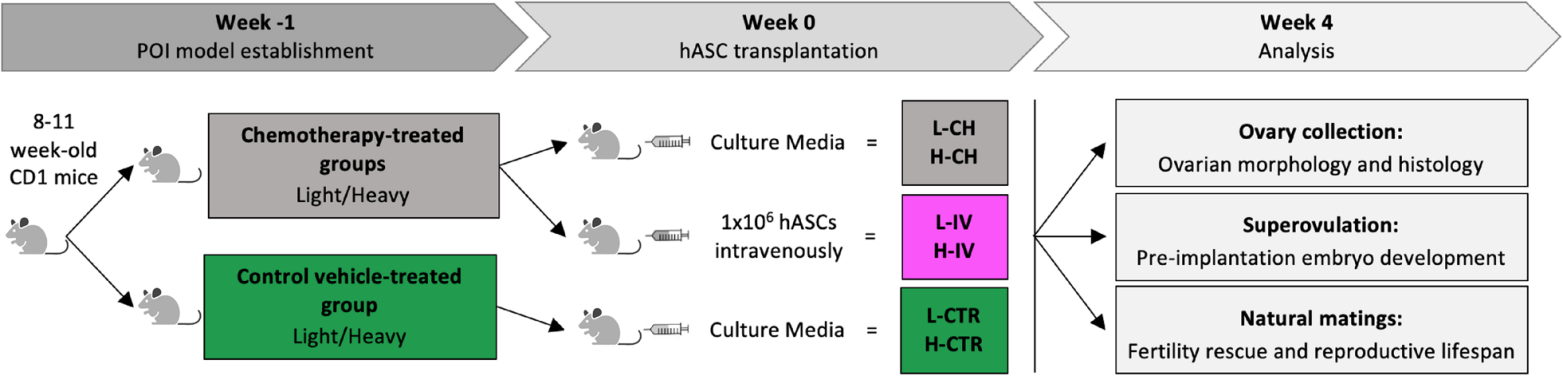
Schematic design of the experiments. For details, see Methods, Animals and treatments paragraph and Results section. L-CH and H-CH: low and heavy chemotherapy-treated group; L-IV and H-IV: intravenously hASC-transplanted groups in low and heavy chemotherapy-treated groups; L-CTR and H-CTR: healthy vehicle-treated control groups for each of the two chemotherapy-treated groups

CD-1 mice were housed in CIMETA (University of Rome Tor Vergata) and mated under standard laboratory conditions in an environmentally controlled room and treated using humane care in order to inflict the least possible pain. All experiments were approved by the Directorate General of Animal Health and Veterinary Medicines (Ministry of Health) with 972/2020-PR authorization and carried out according to the Italian and European rules (D.L. 26/2014; European Directive 2010/63/EU). Two different doses of chemotherapy drugs were used. The light dose (L) consisted of 12 mg/kg busulfan (Sigma-Aldrich) and 120 mg/kg cyclophosphamide (Sigma Aldrich) [21, 42] while the heavy one (H) consisted of 30 mg/kg busulfan (Sigma-Aldrich) and 120 mg/kg cyclophosphamide (Sigma Aldrich) [27, 43]. Busulfan and cyclophosphamide were freshly resuspended in DMSO and then mixed and diluted with saline (Industria Farmaceutica Galenica Senese). For each chemotherapy dose, 8–11 week-old female mice with regular estrous cycle were randomly divided into three groups: healthy control group (L-CTR and H-CTR), chemotherapy-treated group (L-CH and H-CH) and intravenously hASC-transplanted groups (L-IV and H-IV) (Fig. 1). Seven days before transplantation (week -1) CH and IV groups received the respective dose of chemotherapy intraperitoneally, while CTR groups only received the vehicle in which the drugs were resuspended. One day before transplantation hASCs were labelled by incubation with 1 nM LuminiCell Tracker™ 540-cell labelling kit (Millipore) in suspension for 1 h at 37 °C and 5% CO_2_. On the day of transplantation (week 0) mice of all groups were exposed to IR heat lamp for a maximum of 10 min. IV groups then received 1 × 10^6^ cells per mice resuspended in 200 μl of culture media through the tail vein, while CTR e CH groups only received the same amount of culture media. Two IV mice were sacrificed 24 h post-injection. Organs were embedded in OCT (VWR), frozen and cut into 10 μm sections with a cryostat to analyze hASC biodistribution. Images of the LuminiCell Tracker™ derived fluorescence were taken with Leica DMI6000B microscope. Some other mice were sacrificed at week 4 for ovary collection or superovulated for embryo recovery and culture, while the remaining mice were sacrificed at the end of their reproductive life (Fig. 1). Sample sizes are specified along the text and in figure legends. Mice weight was recorded at each time point of the experiment.

### Rejection test

LuminiCell Tracker™ 540 labelled hASCs (1 × 10^6^ cells) were suspended in Geltrex™ (Gibco) and injected subcutaneously. Kodak Image Station In-Vivo FX was utilized to image the mice after anesthetization. Images were taken every 3rd day for 21 days. Regions of Interest (ROIs) were analysed and quantified with Kodak MI Software after subtracting mouse autofluorescence. On day 21 animals were sacrificed, grafts were excised, fixed in 4% PFA, embedded in OCT (VWR) and frozen. Blocks were cut into 10 μm sections with a cryostat for hematoxylin and eosin (H&E) staining and fluorescence analysis. Images were taken with Leica DMI6000B microscope.

### Fertility outcomes

On week 4, mice from every group were superovulated with 10 IU of pregnant mare serum gonadotropin (Folligon, MSD) and 48 h later with 10 IU of human chorionic gonadotropin (Corulon, MSD), followed by mating with age-matched CD1 males. Mated females were sacrificed the day after the vaginal plug (E1.5) to collect 2-cell embryos. Briefly, the oviduct was excised and the ampulla was flushed through the infundibulum with a syringe needle [44]. Preimplantation embryos were then transferred into a culture dish with a pipette and incubated at 37 °C and 5% CO_2_ for 72 h in αMEM supplemented with 2 mM L-glutamine, 100 UI/ml penicillin and 0.1 mg/ml streptomycin, 36 μg/ml sodium pyruvate and 1 mg/ml BSA (all from Sigma-Aldrich) to let them develop into blastocyst. For natural mating, two females were caged with one age-matched CD1 male (trio mating) for eleven months after chemotherapy. After each vaginal plug females were monitored to confirm their pregnancies and to count the number of delivered and healthy pups.

For F1 analysis, body weight was measured when the pups were 21 and 35 days old. Slopes were calculated with Prism. Ovaries were collected and weighted from 35 days old pups.

For F1 fertility analysis, two F1 females for each F0 female were randomly chosen from the first or second litter of the F0 female and mated. At each pregnancy, the number of pups delivered from the two F1 females were averaged. F1 pregnancies were followed for one year.

### Follicle count

Ovaries were collected from each group at week 0 and week 4 and fixed in 4% PFA (KALTEK). After dehydration, tissues were embedded in paraffin and sectioned (5 μm sections) following standard histological procedures. All the follicles from one every six sections were counted after classical H&E staining. Follicles were considered only if the oocyte nucleus was present. They were classified as follows: *primordial* if the oocyte was surrounded, partially or completely, by a layer of squamous granulosa cells; *primary* if the oocyte was surrounded by a single layer of mixed squamous and cuboidal or only cuboidal granulosa cells; *secondary* if the oocyte was surrounded by 2 or more partial or complete layers of granulosa cells; *antral* if an antrum cavity was present. To determine the total number of follicles present in the ovary, the numbers obtained were divided for the number of the ovary sections counted that was then multiplied for the total number of sections obtained from the same ovary [45].

### Immunofluorescence

For IF analysis, antigen retrieval in citrate buffer (pH 6) was performed on sections, obtained as previously described, after deparaffinization and hydration. Sections were permeabilized with PBS-0.01% Triton X for 5 min at room temperature, blocked in 3% BSA for 30 min at room temperature and then incubated with αSMA primary antibody (A2547, Sigma-Aldrich) (1:250) in 0.3% BSA overnight at 4 °C.Primary antibody binding was detected incubating section with Alexa Fluor 568-labeled goat anti-mouse secondary antibody (Thermo Fisher Scientific) (1:500) for 1 h at room temperature. Hoechst was used as counterstain. Sections were mounted in PBS-glycerol (1:1) and images were taken with Leica DMI6000B microscope. MicroVascular Density (MVD) analysis of whole hilum sections was performed with ImageJ software (NIH).

### Statistical analysis

Data collected were analyzed with GraphPad Prism (software version 7.0, San Diego, CA). Results were given as mean ± SD and *p* value was determined by one-way or two-way Anova and Bonferroni post-analyses or t-test. Statistical significance was based on *p* value: *p* < 0.05, *p* < 0.01, *p* < 0.001 and *p* < 0.0001 are indicated with *a*, *b*, *c* and *d* or with *, **, *** and **** if significance is calculated vs CTR or CH groups, respectively.

## Results

### hASCs isolation and characterization

**Fig. 2:**
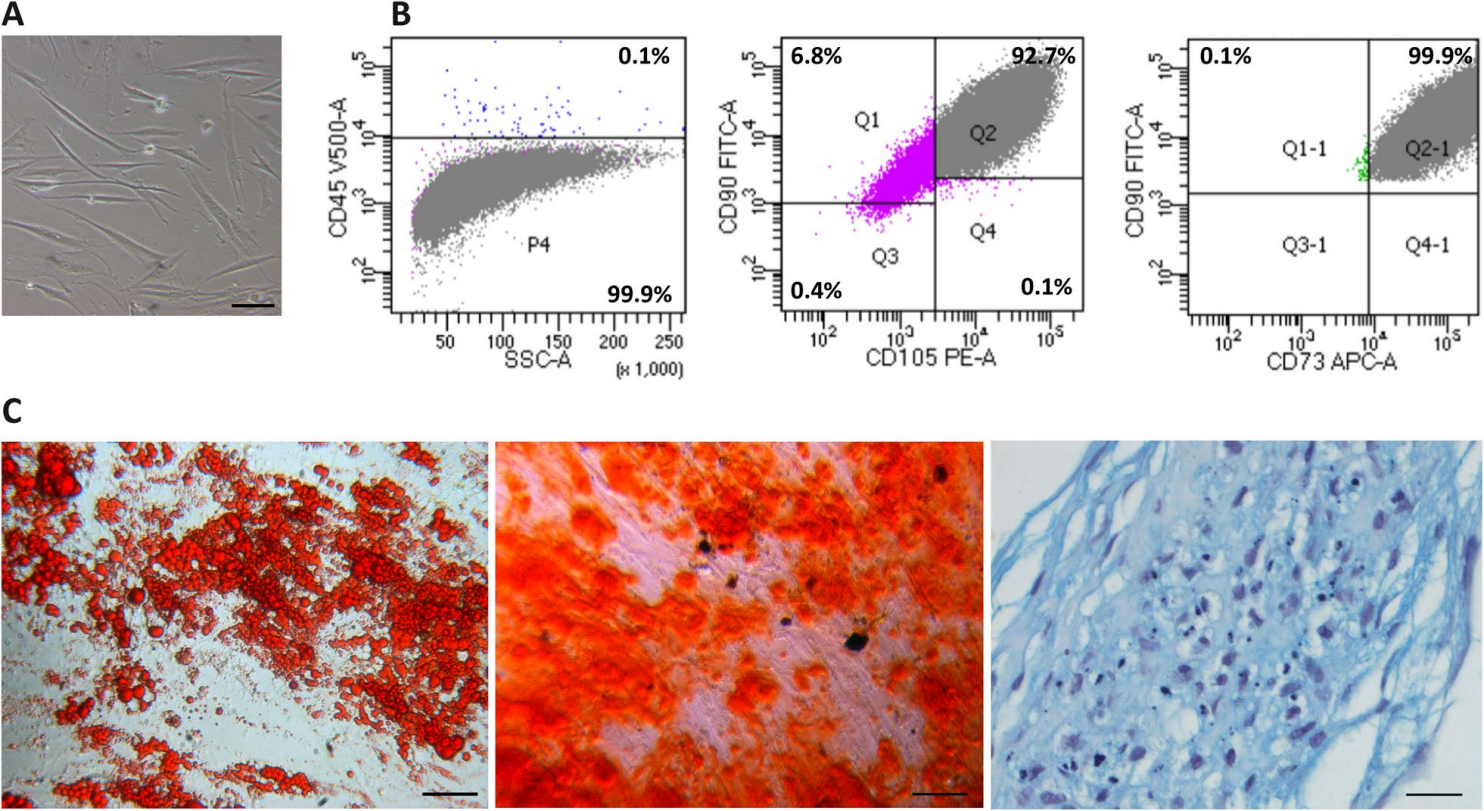
Characterization of hASCs. **A** Phase contrast micrograph of in vitro cultured cells isolated from the adipose tissue biopsies. Scale bar = 100 μm. **B** Cytofluorimetric analyses showing absence of CD45 (left) and positivity for CD105, CD90 and CD73 (centre and right) in the putative hASCs obtained in culture. **C** Trilineage mesenchymal differentiation potential of hASCs into adipocytes (left), osteoblast (centre) and chondroblast (right), as confirmed through staining of lipid droplets with Oil Red O, mineralized matrix deposition with Alizarin Red S and sulphated proteoglycan deposition with Alcian Blue, respectively. Scale bar left and centre = 100 μm, scale bar right = 25 μm

Lipoaspirate samples were enzymatically digested overnight. The SVF was then plated to obtain primary cell lines. At passage 3, under a phase contrast microscope, most of the cells appeared as a heterogeneous population of mostly fusiform and elongated cells (Fig. 2A). Their identity as hASCs was determined on the basis of the minimal criteria established for MSCs by the International Society for Cellular Therapy [46]: (i) strong adherence to tissue-culture plastic (Fig. 2A), (ii) > 90% positivity for cell markers CD105, CD73 and CD90 and negativity for the leukocyte marker CD45 (99,9%) evaluated by flow cytometric analysis (Fig. 2B), and (iii) capability to differentiate in vitro into adipocytes, osteoblasts and chondroblasts following appropriate differentiation protocols (Fig. 2C).

### hASCs tracking and distribution in the host organs

To verify that the transplanted hASCs would not be rejected by the hosting mice, we performed a rejection test by subcutaneously injecting hASCs labelled with biocompatible organic fluorescent nanoparticles (LuminiCell Tracker™ 540) (Additional file 2: Figure S1A) encapsulated in Geltrex. In vivo imaging revealed that fluorescent cell aggregates remained in the transplant area up to three weeks undergoing a progressive fluorescence intensity decrease (Additional file 2: Figure S1B). Day 21 histological sections showed that along this period the grafted cells retained detectable fluorescence indicative of viability (Additional file 2: Figure S1C).

Tracking the distribution of the transplanted labelled hASCs in the host’s organs after tail vein injection, we observed that 24 h after transplantation numerous fluorescent cells were in the lungs and some in all other analysed organs including kidneys, liver and ovaries. Four weeks post-injection, only a few fluorescent cells were found scattered throughout ovarian tissues (Additional file 3: Figure S2).

### hASC transplantation alleviates morphological ovarian changes induced by chemotherapy

Given the variety of chemotherapeutic treatments used in literature to induce POI in female mice, to establish more precisely the efficacy of hASC systemic transplantation in preserving the ovaries from the gonadotoxic side effect of chemotherapy, we sought to compare two models of POI with different severity: one defined as light (L), via administration of 120 mg/kg Cy + 12 mg/kg Bu intraperitoneally (ip) [21, 42], the other termed heavy (H) and characterized by ip injection of 120 mg/kg Cy + 30 mg/kg Bu [27, 43]. In both cases, hASCs were injected through the tail vein one week after (w0) chemotherapeutic treatments (IV groups) (Fig. 1).

**Fig. 3:**
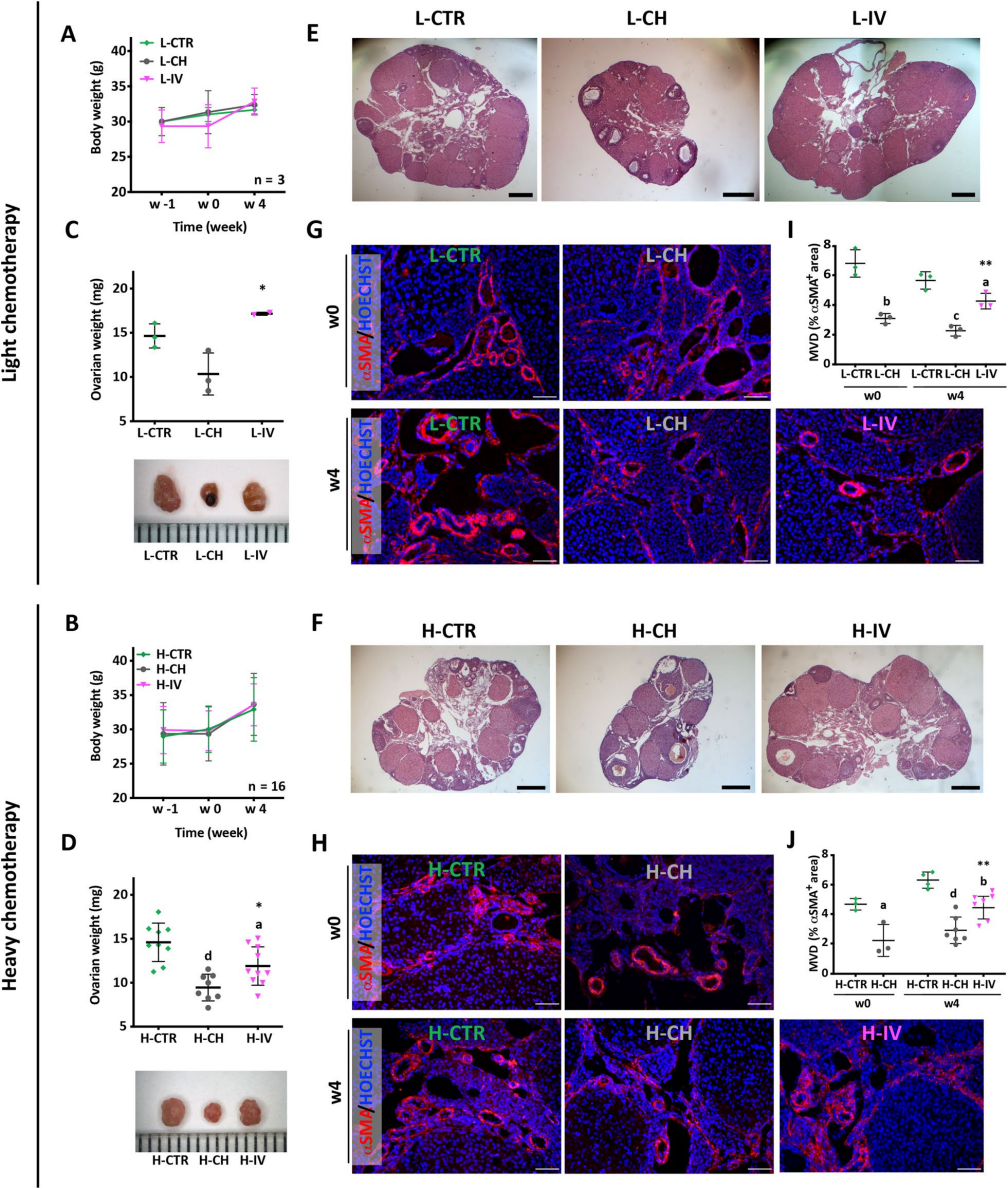
Effects of chemotherapy and hASC transplantation on body weight and ovarian morphological features. **A, B** Body weight of CTR, CH and IV females, following both light (L) and heavy (H) chemotherapeutic treatments at the indicated time points; sample size: n = 3 and n ≥ 16, respectively. Values are expressed as mean ± SD. **C, D** **top panel** Ovarian weight at week 4. Values are displayed as scattered dot plot showing mean ± SD. Statistical significance is calculated with One-Way ANOVA vs CTR (letters) and vs CH (asterisks) as described in “Methods” section. Sample size: n = 3 and n ≥ 8 for light (L) and heavy (H) chemotherapeutic treatment, respectively. **C, D** **bottom panel** Representative micrographs of ovaries at week 4. Distance between ruler ticks = 1 mm. **E, F** Representative midsagittal H&E stained sections of w4 ovaries showing the hilum region. Scale bar = 500 μm. L-IV and H-IV images are combined because histological sections exceeded camera field of view. **G, H** Representative IF images of the hilum region following αSMA^+^ staining. Scale bar = 50 μm. **I, J** Quantification of the MVD as the percentage of αSMA^+^ area compared to the area of the whole section. Values are displayed as scattered dot plot showing mean ± SD. Statistical significance is calculated with t-test (w0) and with One-way ANOVA (w4) vs CTR (letters) and vs CH (asterisks) as described in "Methods". Sample size: n = 3 and n ≥ 3 for light (L) and heavy (H) chemotherapeutic treatment, respectively

Since chemotherapy-induced POI is often associated with body weight loss, control and treated mice were weighted up to 4 weeks after chemotherapy treatments. While the body weight curves of the mice of all groups overlapped throughout the analysed period (Fig. 3A, B), at week 4, the ovary weights of chemotherapy-treated animals resulted significantly lower than controls, as expected; interestingly such effect was almost completely prevented following hASC transplantation (Fig. 3C, D). Histological analyses of ovaries revealed that in females treated with either chemotherapy protocols, the evident reduction of size was associated with a more compact stroma and a smaller hilum (a ligament-like structure between the ovary and the reproductive tract) (Fig. 3E, F). In addition, IF analysis for αSMA (a marker of the vascular wall smooth muscle cells), showed that both chemotherapy treatments caused a severe reduction of the percentage of the ovarian vascularized area measured at w0 (Fig. 3G–H). Interestingly at w4, both morphological parameters were significantly improved by hASC transplantation in comparison to CH groups (Fig. 3E–J).

### hASC transplantation prevents the progressive ovarian reserve depletion caused by chemotherapy

**Fig. 4:**
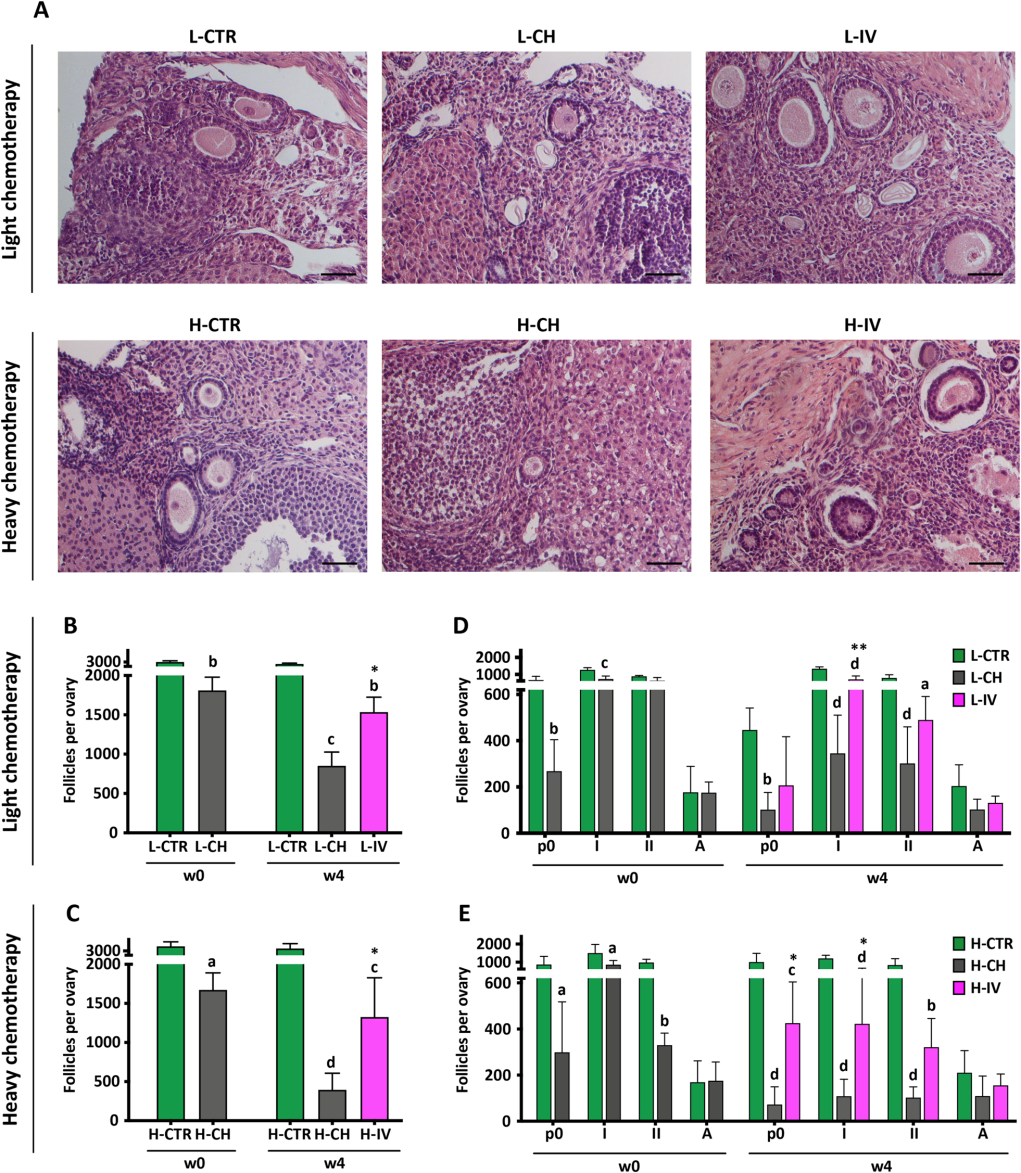
Follicle morphology, count and classification. **A** Representative micrographs of different categories of follicles containing oocyte present in sections of ovaries obtained from animals subjected to the indicated treatments. Scale bar = 50 μm. **B, C** Total number of follicles per ovary. Values are expressed as mean ± SD. Significance is calculated with t-test (w0) and with One-way ANOVA (w4) vs CTR (letters) and vs CH (asterisks) as described in "Methods". **D, E** Number of follicles per ovary in function of the maturation stage: primordial (p0), primary (I), secondary (II) and antral (A). Values are expressed as mean ± SD. Significance is calculated with Two-way ANOVA vs CTR (letters) and vs CH (asterisks) as described in "Methods". Sample size: n ≥ 3 and n ≥ 4 for light (L) and heavy (H) chemotherapeutic treatment, respectively

To evaluate the extent of the damage caused by the two doses of chemotherapy, we then performed an accurate evaluation of ovarian reserve through follicle classification and count, as shown in Fig. 4. The analysis was performed by observing histological sections (Fig. 4A) and determining follicle number with a procedure specified in "Methods". At the time of hASC injection (w0) ovaries from both chemotherapy protocols presented almost half of the total number of follicles compared to CTR (L-CH: 1810 ± 377.2 follicles vs L-CTR: 3004.0 ± 238.3 follicles, *p* < 0.01, n ≥ 3; H-CH: 1672.0 ± 218.5 follicles vs H-CTR: 3523.0 ± 1058.0 follicles, *p* < 0.05, n = 4). At week 4, the total number of follicles was further reduced in a dose-dependent manner to about 30% and 12% of the CTR, in L-CH and H-CH, respectively (L-CH: 851.5 ± 349.7 follicles vs L-CTR: 2778.0 ± 162.9 follicles, *p* < 0.001, n ≥ 3; H-CH: 393.7 ± 212.5 follicles vs H-CTR: 3272.0 ± 563.8 follicles, *p* < 0.01, n ≥ 4). In both chemotherapy protocols, hASC injection was able to consistently arrest such further follicle decrease (L-IV: 1536.0 ± 377.5 follicles, p < 0.05, n = 4; H-IV: 1325.0 ± 502.5 follicles, *p* < 0.05, n ≥ 4) (Fig. 4B, C). Based on follicle classification, we observed that primordial and primary follicles were the most affected by either CH protocols as well as protected by hASC transplantation (L-IV: 707.5 ± 202.4 primary follicles vs L-CH: 345.2 ± 165.2 primary follicles, *p* < 0.01, n = 4; H-IV: 425.5 ± 178.8 primordial follicles vs H–CH: 72.7 ± 76.5 primordial follicles, *p* < 0.05; H-IV: 422.5 ± 246.2 primary follicles vs H-CH: 108.8 ± 73.1 primary follicles, *p* < 0.05; n ≥ 4) (Fig. 4D, E).

### hASC transplantation preserves the oocyte capability to sustain pre-implantation embryo development and delays chemotherapy-induced POI onset

To evaluate the quality of the surviving oocytes in CH and IV groups, we tested their capability, after fertilization, to sustain normal pre- and post-implantation embryo development in vitro and in vivo, respectively.

**Fig. 5:**
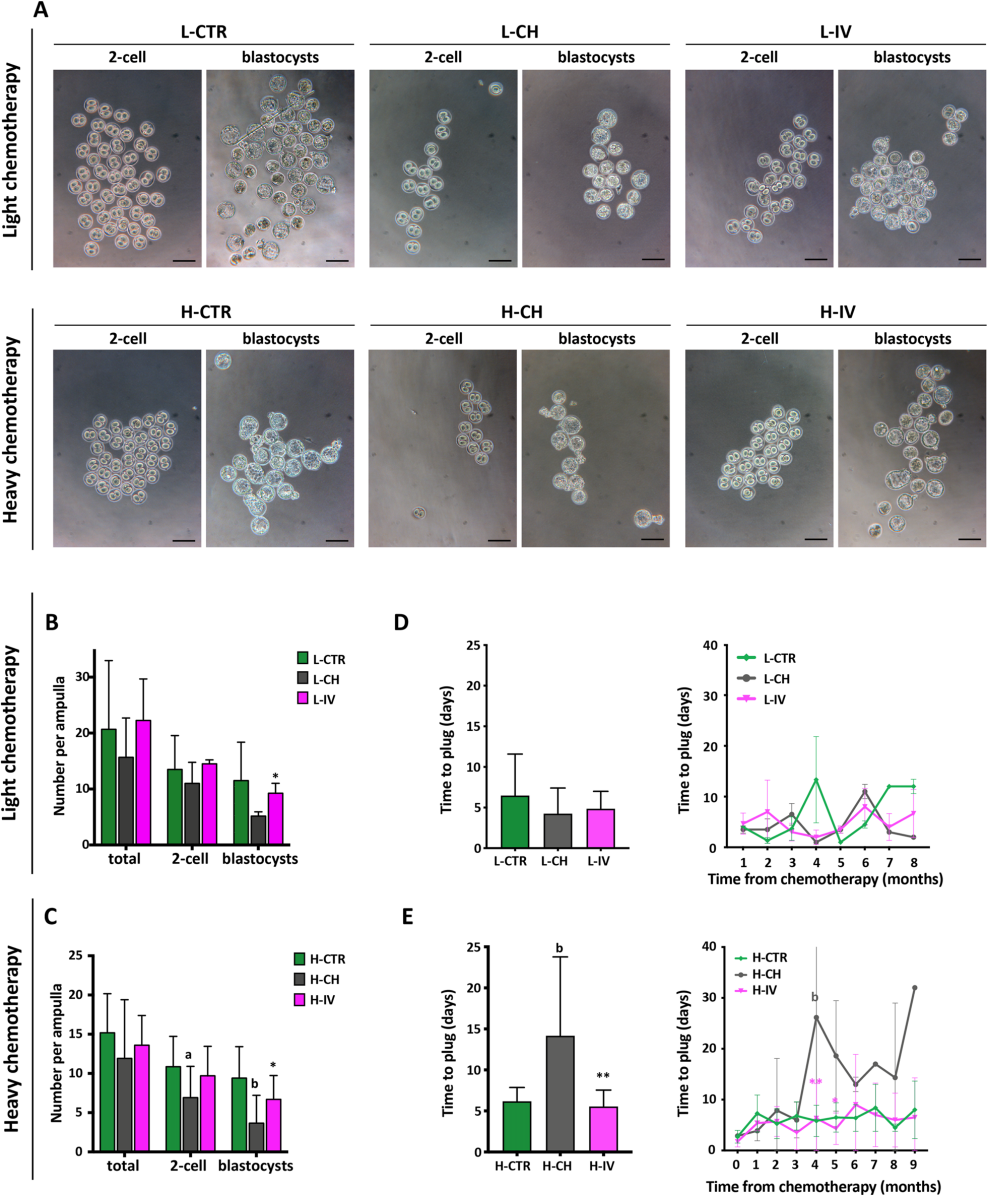
Analysis of pre-implantation embryo development in vitro and of time to plug after natural mating. **A, B, C** After superovulation, females were mated, sacrificed at E1.5 and embryos collected from both ampullae were counted and classified. Two-cell embryos were cultured in vitro for 72 h to reach the blastocyst stage as seen in the representative micrographs in **A** (Scale bars = 100 μm). In **B** and **C** values per ampulla are expressed as means ± SD. Significance is calculated with t-test vs CTR (letters) and vs CH (asterisks) as described in "Methods". Sample size: n = 3 and n ≥ 10 for light (L) and heavy (H) chemotherapeutic treatment, respectively. **D, E** Days of natural mating required to achieve a vaginal plug. Mean values for the whole period analysed are shown on the left panels, while the trend of the mean values over time is shown on the right panels. Values are expressed as means ± SD. Significance is calculated with One and Two-way ANOVA, respectively, vs CTR (letters) and vs CH (asterisks) as described in "Methods". Sample size: n = 3 and n ≥ 7 for light (L) and heavy (H) chemotherapeutic treatment, respectively

To investigate pre-implantation development, at w4 after chemotherapy, mice from all experimental groups were super ovulated and mated with males of proven fertility. The plugged females were sacrificed at 1.5 day post coitum. At this time, the ovulation performance did not appear to be affected by the chemotherapy. In fact, despite the marked differences in the ovary stockpile reported above, females of all groups ovulated similar numbers of oocytes as estimated by counting the total numbers of degenerating/fragmented or unfertilized oocytes and two-cell embryos recovered from the ampulla. The number of 2-cell embryos was, however, always lower in CH females in comparison to those of CTR and IV groups, although the differences among groups were not statistically significant except for H-CH *versus* H-CTR. Noteworthy, both chemotherapy protocols caused a significant reduction of the capability of 2-cell embryos to develop in vitro to the blastocyst stage while hASC injection preserved such capability (Fig. 5A–C).

To investigate post-implantation development and pregnancy outcomes, control and treated females were allowed to mate with males to monitor various parameters of the reproductive life up to 11 months after hASC transplantation. Interestingly, only H-CH females showed a significant increase in the mean number of days of mating required before becoming pregnant, which was completely prevented in H-IV females (H–CH: 14.2 ± 9.6 vs H-CTR: 6.2 ± 1.7, H-IV: 5.5 ± 2.0, *p* < 0.01, n = 10) (Fig. 5D, E).

**Fig. 6:**
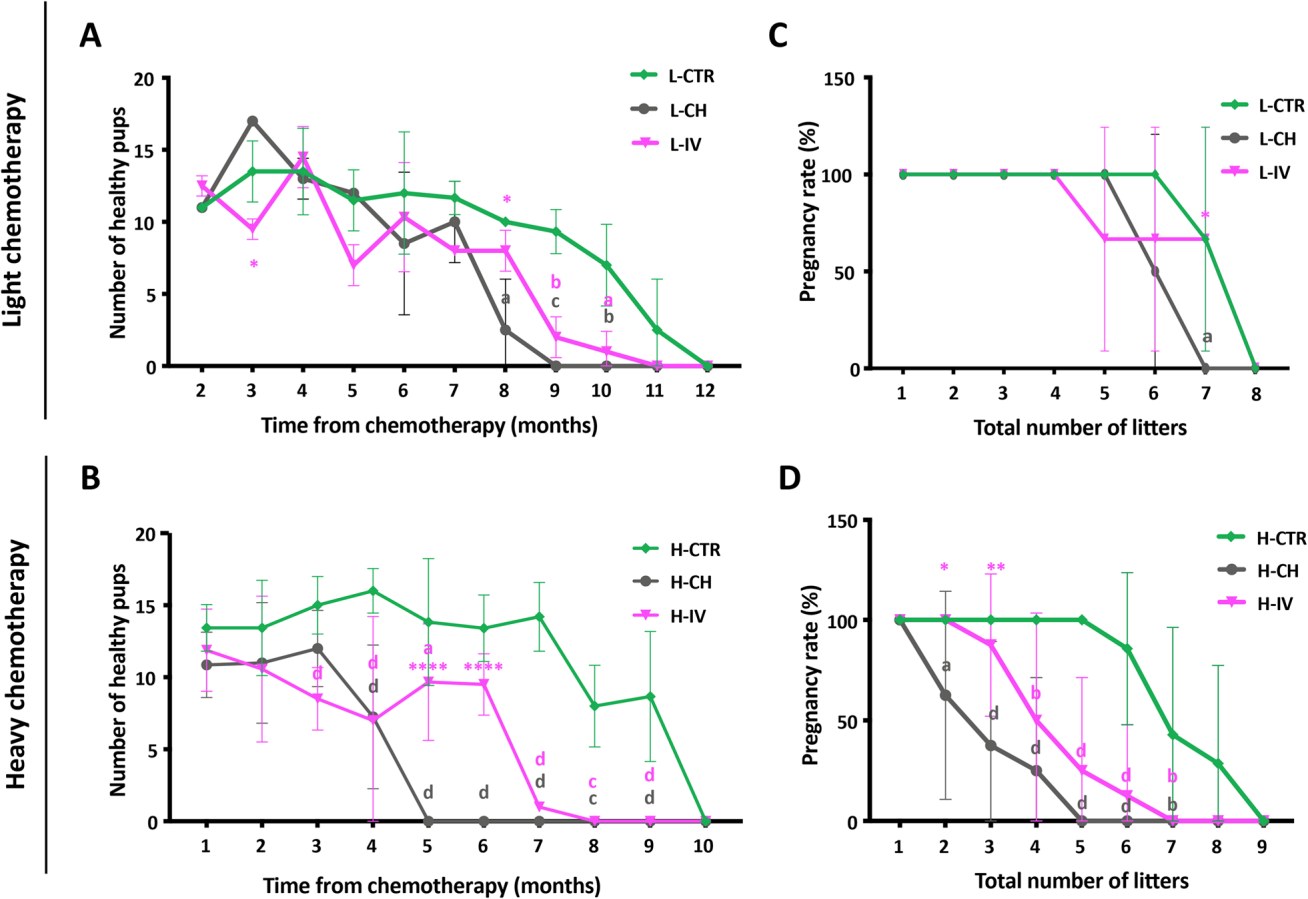
Analysis of mice fertility after natural mating. **A, B** Number of healthy pups delivered by females at each month along their reproductive lives. Values are expressed as means ± SD. Significance is calculated with Two-way ANOVA vs CTR (letters) and vs CH (asterisks) as described in "Methods". Sample size: n = 3 and n ≥ 7 for light (L) and heavy (H) chemotherapeutic treatment, respectively. **C, D** Percentage of females that became pregnant and delivered the indicate number of litters. Values are expressed as means ± SD. Significance is calculated with Two-way ANOVA vs CTR (letters) and vs CH (asterisks) as described in "Methods". Sample size: n = 3 and n ≥ 7 for light (L) and heavy (H) chemotherapeutic treatment, respectively

As expected, with age, all groups showed a progressive decrease in the number of healthy pups delivered (Fig. 6A, B). It was evident, however, that L-CH females lost fertility more rapidly than those of either L-CTR or L-IV groups and that such condition was exacerbated in H-CH females compared to H-CTR and H-IV groups. On the other hand, hASC transplantation was able to delay the onset of such chemotherapy-induced POI. This was especially evident in the H-CH condition in which hASC transplantation was able to significantly prolong mice reproductive lifespan (H-IV: 4.1 ± 1.7 vs H-CH: 2.4 ± 1.4 months, *p* < 0.05, n = 8), allowing more than 25% of females to keep delivering a mean of 9 pups per litter for two more months compared to H-CH group (Fig. 6A, B and Table 1, Additional file 1: S1). Moreover, hASC transplantation was able to alleviate POI severity. Indeed, it increased the mean number of litters delivered throughout the female reproductive lifespan (H-IV: 3.8 ± 1.3 litters vs H-CH: 2.3 ± 1.3 litters, *p* < 0.05, n = 8). In particular, only 62.5% of H-CH females were able to deliver up to 2 litters compared to the 100% of H-IV and H-CTR ones (*p* < 0.05, n ≥ 7); only 37.5% of H-CH females were able to deliver up to 3 litters compared to the 87.5% of H-IV ones (p < 0.01, n = 8); none of the H-CH females could reach 5 or 6 pregnancies while 25% and 12.5% of H-IV, respectively, did so (Fig. 6C, D and Table 1).

**Table 1:** Summary of fertility recovery study

	H-CTR	H-CH	H-IV
Number of animals	7	8	8
Total number of pups	88.9 ± 20.6	24.5 ± 17.3 **d** ^1^	36.3 ± 19.2 **d** ^1^
Number of pups/litter (months 1–4)	14.5 ± 1.3	10.3 ± 2.1 **a** ^1^	9.5 ± 2.2 **b** ^1^
Number of litters	6.6 ± 1.1	2.3 ± 1.3 **d** ^1^	3.8 ± 1.3 **c** ^1^, * ^2^
Months of fertility from w-1	7.9 ± 1.2	2.4 ± 1.4 **d** ^1^	4.1 ± 1.7 **c** ^1^, * ^2^
Pregnancy rate (up to 1 litter)	100% (7/7)	100% (8/8)	100% (8/8)
Pregnancy rate (up to 2 litters)	100% (7/7)	62.5% (5/8) **a** ^1^	100% (8/8) * ^1^
Pregnancy rate (up to 3 litters)	100% (7/7)	37.5% (3/8) **c** ^1^	87.5% (7/8) ** ^1^
Pregnancy rate (up to 4 litters)	100% (7/7)	25% (2/8) **d** ^1^	50% (4/8) **b** ^1^
Pregnancy rate (up to 5 litters)	100% (7/7)	–	25% (2/8) **d** ^1^
Pregnancy rate (up to 6 litters)	85.7% (6/7)	–	12.5% (1/8) **d** ^1^
Pregnancy rate (up to 7 litters)	42.9% (3/7)	–	–
Pregnancy rate (up to 8 litters)	28.6% (2/7)	–	–
Pregnancy rate (up to 9 litters)	–	–	–

^1^Significance is calculated with One-way ANOVA vs CTR (letters) and vs CH (asterisks) as described in "Methods" section

^2^Significance is calculated with t-test vs CH (asterisks) as described in "Methods" section

We also monitored the body growth, ovarian weight, and fertility after natural mating of F1 pups without finding any significant difference among groups. Interestingly, 1 out of 8 H-CH F1 pups resulted completely sterile (Additional file 4: Figure S3).

## Discussion

MSC transplantation has been recently proposed as a possible treatment to improve the quality of life in patients forced to face the consequences of oncologic treatments. MSCs hold great promise in the field of regenerative medicine because of their ability to repair tissues mainly secreting various growth factors, cytokines and chemokines that decrease local inflammation [47]. Several studies have recently shown that in situ or systemic injection of MSCs of various animal species, and from different anatomical sources, is able to restore impaired ovarian functions in animal models of POI [18, 19]. However, to our knowledge, none of these studies investigated whether human ASC transplantation was actually able to slow down POI onset in terms of infertility insurgence. In addition, all studies differ in the animal model established due to the variety of chemotherapeutic treatments, making it hard to compare and to understand the extent of the efficacy of MSC treatment. For these reason we decided to carry out a long-term study, monitoring the reproductive lifespan in order to verify if human ASC transplantation can delay or prevent chemotherapy-derived POI onset. We chose outbred CD1 mice as animal model to more closely match the response of a human population to chemotherapeutic agents and transplantation, and to produce data that have a greater translational value compared to those obtained in inbred strains. Moreover, we administered two different doses of chemotherapy, already used in literature to induce POI [27, 42], in order to understand what degree of ovarian insufficiency they cause and to compare the efficacy of ASC transplantation in both cases.

We isolated putative hASCs and confirmed their identity according to cytofluorometric analysis and the three-lineage differentiation test. Then, we verified that after transplantation these cells were not rapidly rejected and were distributed in various host organs, including ovaries. The observation that most of hASC remained entrapped into the lungs as previously reported [48–50], and that only a few were traceable in other sites, support the notion that their ability to repair injured tissues is mainly due to the systemic release of trophic factors rather than to their differentiation into multiple cell types [23, 25, 28, 29, 31, 34, 51].

The first indication that hASC transplantation can alleviate the deleterious effects of chemotherapy on the ovaries was the recovery of almost normal weight and size of this organ in IV female groups. This effect was associated with improvement of histological ovarian features including stroma, vascularization, and follicle population. In the latter case, both chemotherapeutic treatments appeared to affect mainly primordial and primary follicles with a kinetics that was similar at w0 but more severe for H-CH at w4. Strikingly, in both cases, hASC transplantation was able to significantly arrest the progressive depletion of such follicles following chemotherapy. This suggests that systemic hASC transplantation is able to readily establish an ovarian environment able to maintain follicle survival under unfavorable conditions and perhaps allowing follicular cells and/or the oocytes to repair DNA damage induced by chemotherapy that can trigger apoptosis [52]. This also implies that earlier hASC transplantation could more efficiently protect the ovarian reserve from chemotherapy. In line with these results, it was reported that hASC transplantation, even without chemotherapy, was able to slow down naturally occurring oocyte and ovarian aging [53].

Starting at w4 after transplantation, although CH females and those receiving hASC showed ovulation performance and fertility rate essentially similar to untreated controls, the competence of their embryos to develop in vitro into blastocysts was significantly lower in the 2-cell embryos obtained from CH females in comparison to both CTR and IV females. This indicates that both chemotherapy protocols can cause permanent damage in surviving oocytes compromising their capability to sustain normal preimplantation embryo development while hASC transplantation defines intraovarian conditions allowing oocytes to recover such competence.

The mating experiments revealed the effectiveness of the CH treatments to induce POI with different efficacy and of hASC transplantation to delay its onset and severity. In this regard, we found that L-CH and H-CH females lost fertility about three and five months before their controls, respectively. On the other hand, hASC transplantation was able to delay the onset of chemotherapy-induced POI. In fact, in the H-IV group, more than 25% of females kept delivering a mean of 9 pups per litter for two more months and also increased the mean number of litters delivered throughout their reproductive lifespan compared with the females that only received chemotherapy.

In addition, H-CH females showed a significant increase in the mean number of days required to become pregnant, which was completely prevented by transplantation. As discussed above regarding oocyte competence, also in this case hASC transplantation might preserve the ovarian microenvironment necessary to allow a normal estrous cycle and, therefore, physiological reproductive functions. This is supported as well by the evidence coming from other works that ASCs decrease stroma and granulosa cell apoptosis [23, 51]. In this regard, L-CH did not seem sufficient to induce a complete POI in our system. In fact, there was no difference between control and treated groups in the number of mating days required to achieve pregnancy throughout the time analyzed, suggesting the maintenance of a normal estrous cycle in the L-CH-treated group.

Finally, the observation that 1 out of the 8 H-CH F1 females monitored for reproductive performance during their life, resulted completely sterile suggests the possibility of transgenerational inheritance of the CH-induced ovary alterations not observed in the H-IV F1 female group. However such a possibility needs to be supported by more observations also including later generations [37].

On the basis of the present data and the many trials that are already using hASCs for diverse clinical applications, it appears quite feasible in a near future their use to the bedside as autologous cells to recover gonad toxicity. Moreover, since MSC regenerative potential seems to be mainly related to secreted factors, it will be important to investigate whether the same results can be obtained by using conditioned media or extracellular vesicles/exosomes produced by hASCs, as recently suggested [51, 54, 55].

## Conclusions

In conclusion, we obtained convincing evidence that hASC intravenous transplantation can alleviate the deleterious effects of chemotherapy on ovarian reserve, on the quality of oocytes and their competence to develop, as well as on ovarian functionality and reproductive performance. We demonstrated for the first time that hASC transplantation is actually able to prolong reproductive lifespan in both mild and severe POI models. Hopefully this would result in counteracting chemotherapy-induced infertility in women, while also postponing the insurgence of menopause and its related symptoms. Such possible translation to the clinic is strengthened by the fact that we used an outbred mouse strain, which better resembles human population diversity.

## Supplementary Information

**Additional file 1**.

**Additional file 2**: **Figure S1**. hASC rejection test. **A** Micrograph of hASCs after labelling with LuminiCell Tracker™ 540 (AIE). Scale bar = 100 μm. **B** In vivo monitoring of fluorescent hASCs after Geltrex encapsulation and subcutaneous injection.Visualization by Kodak Image Station In-Vivo FX. Dotted black circle indicates the ROI (Region of Interest) used for fluorescence quantification of the graft. XGF = xenograft fluorescence, MAF = mouse autofluorescence. **C** Two consecutive sections of the graft (top panel stained with H&E, bottom panel stained with Hoechst) showing viable cells retaining AIE fluorescence. Scale bars = 75 μm. 

**Additional file 3**: **Figure S2**. hASC tracking. Representative micrograph of cryostat sections of the indicated organs showing hASC distribution 24h (**A**) and 4 weeks (**B**) post intravenous injection. The presence of hASCs is evidenced by the fluorescent dots (arrowheads). Scale bars = 75 μm.

**Additional file 4**: **Figure S3**. F1 study. **A** Slope of the growth curve of F1 females. Body weight was measured at day 21 and 35. Values are expressed as means±SD. Sample size: n≥3 where n is the number of litters analysed. **B** Weight of the ovaries from 35-day-old F1 females. Values are expressed as means±SD. Sample size: n≥3 where n is the number of litters analysed. **C **Number of F2 pups delivered from F1 females. Mean values for the whole period analysed are shown on the left panel, while the trend of the mean values overtime is shown on the right panel. Values are expressed as means±SD. H-CTR: n=3, H-CH: n=4, H-IV: n=4 where n is the number of F0 females analysed, for each of which data from two F1 females were averaged.

## Data Availability

All data generated or analyzed during this study are included in this published article (and its supplementary information files). Data sharing is not applicable to this article as no datasets were generated or analyzed during the current study.

## Abbreviations

ASCs: Adipose-derived Stromal Cells
hASCs: Human Adipose-derived Stromal Cells
MSCs: Mesenchymal stromal cells
POI: Premature ovarian insufficiency
H&E: Hematoxylin and eosin
αSMA: Alpha smooth muscle actin
FMR1: Fragile X mental retardation 1
GnRH-a: Gonadotropin-releasing hormone agonist
FSH: Follicle stimulating hormone
FSHR: Follicle stimulating hormone receptor
AMH: Anti-Müllerian hormone
E2: Estradiol
αMEM: Alpha minimum essential medium
FBS: Fetal bovine serum
PBS: Phosphate-buffered saline
SVF: Stromal vascular fraction
IBMX: 3-Isobutyl-1-methylxanthine
TGF- β1: Transforming growth factor beta 1
CD: Cluster of differentiation
7-AAD: 7-Aminoactinomycin D
ROI: Region of Interest
PFA: Paraformaldehyde
OCT: Optimal cutting temperature
BSA: Bovine serum albumin
MVD: MicroVascular density
AIE: Aggregation-induced emission
XGF: Xenograft fluorescence
MAF: Mouse autofluorescence
