# Supplementary material for: Human adipose-derived stromal cells transplantation prolongs reproductive lifespan on mouse models of mild and severe premature ovarian insufficiency
Source: Stem Cell Res Ther. 2021 Oct 10;12:537. doi: 10.1186/s13287-021-02590-5 (PMC8504050; doi:10.1186/s13287-021-02590-5)
Supplement: Supplementary file 1 — Additional file 1. [file 13287_2021_2590_MOESM1_ESM.docx]

| **Months from chemotherapy** | **1** | **2** | **3** | **4** | **5** | **6** | **7** | **8** | **9** | **10** |
| --- | --- | --- | --- | --- | --- | --- | --- | --- | --- | --- |
| **H-CTR females delivering pups**   - **Number/total** - **%** | 7/7  100 | 7/7  100 | 5/7  71.4 | 6/7  85.7 | 6/7  85.7 | 5/7  71.4 | 5/7  71.4 | 2/7  28.6 | 3/7  42.9 | -  - |
| **H-CH females delivering pups**   - **Number/total** - **%** | 7/8  87.5 | 5/8  62.5 | 3/8  37.5 | 3/8  37.5 | -  - | -  - | -  - | -  - | -  - | -  - |
| **H-IV females delivering pups**   - **Number/total** - **%** | 8/8  100 | 7/8  87.5 | 6/8  75 | 3/8  37.5 | 3/8  37.5 | 2/8  25 | 1/8  12.5 | -  - | -  - | -  - |

**Table S1.** **Data related to Figure 5B**
