# Supplementary figures and images for: Human adipose-derived stromal cells transplantation prolongs reproductive lifespan on mouse models of mild and severe premature ovarian insufficiency
Source: Stem Cell Res Ther. 2021 Oct 10;12:537. doi: 10.1186/s13287-021-02590-5 (PMC8504050; doi:10.1186/s13287-021-02590-5)

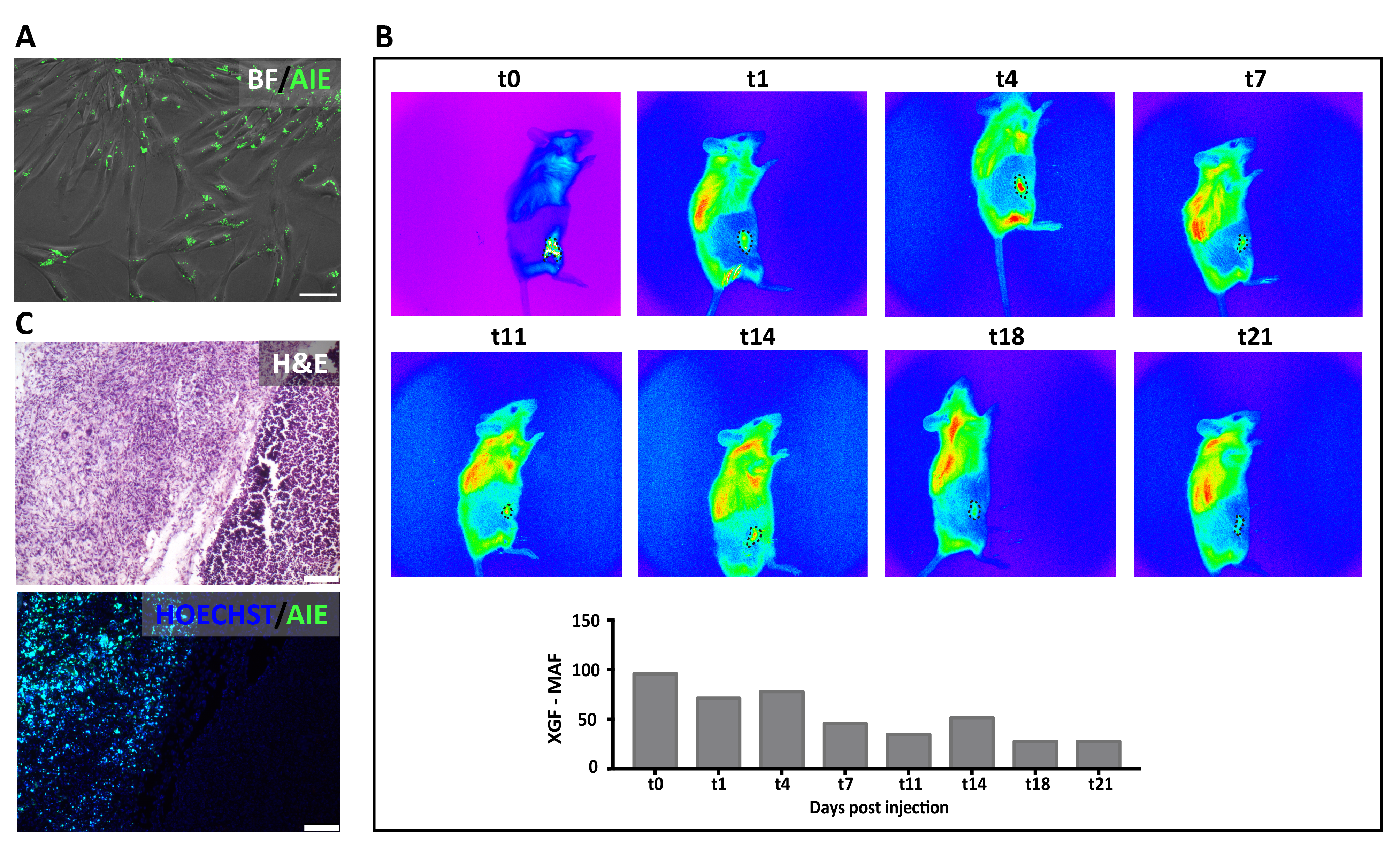

Supplement: Supplementary file 2 — Additional file 2: Figure S1. hASC rejection test. A Micrograph of hASCs after labelling with LuminiCell Tracker™ 540 (AIE). Scale bar = 100 μm. B In vivo monitoring of fluorescent hASCs after Geltrex encapsulation and subcutaneous injection.Visualization by Kodak Image Station In-Vivo FX. Dotted black circle indicates the ROI (Region of Interest) used for fluorescence quantification of the graft. XGF = xenograft fluorescence, MAF = mouse autofluorescence. C Two consecutive sections of the graft (top panel stained with H&E, bottom panel stained with Hoechst) showing viable cells retaining AIE fluorescence. Scale bars = 75 μm. [file 13287_2021_2590_MOESM2_ESM.png]

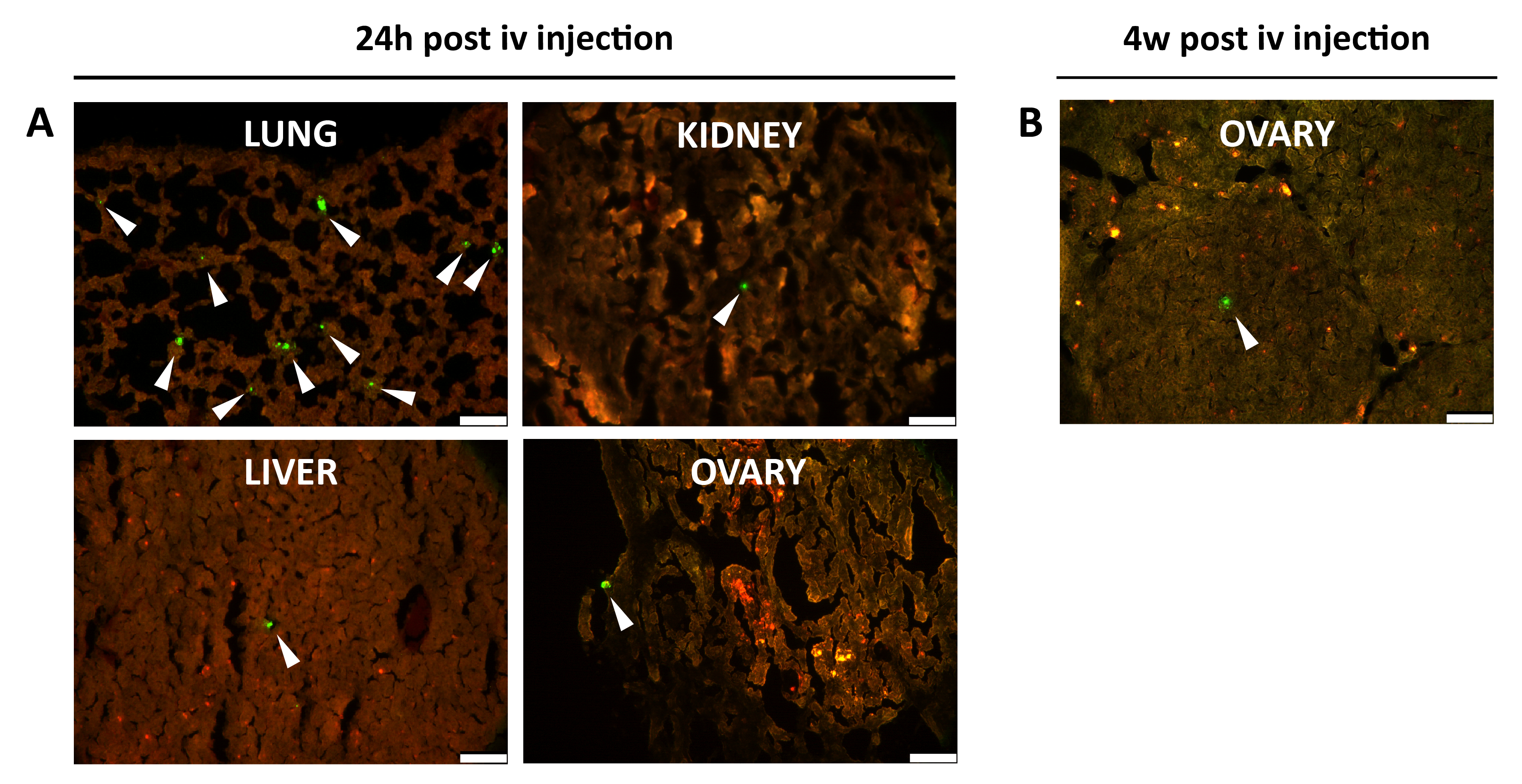

Supplement: Supplementary file 3 — Additional file 3: Figure S2. hASC tracking. Representative micrograph of cryostat sections of the indicated organs showing hASC distribution 24h (A) and 4 weeks (B) post intravenous injection. The presence of hASCs is evidenced by the fluorescent dots (arrowheads). Scale bars = 75 μm. [file 13287_2021_2590_MOESM3_ESM.png]

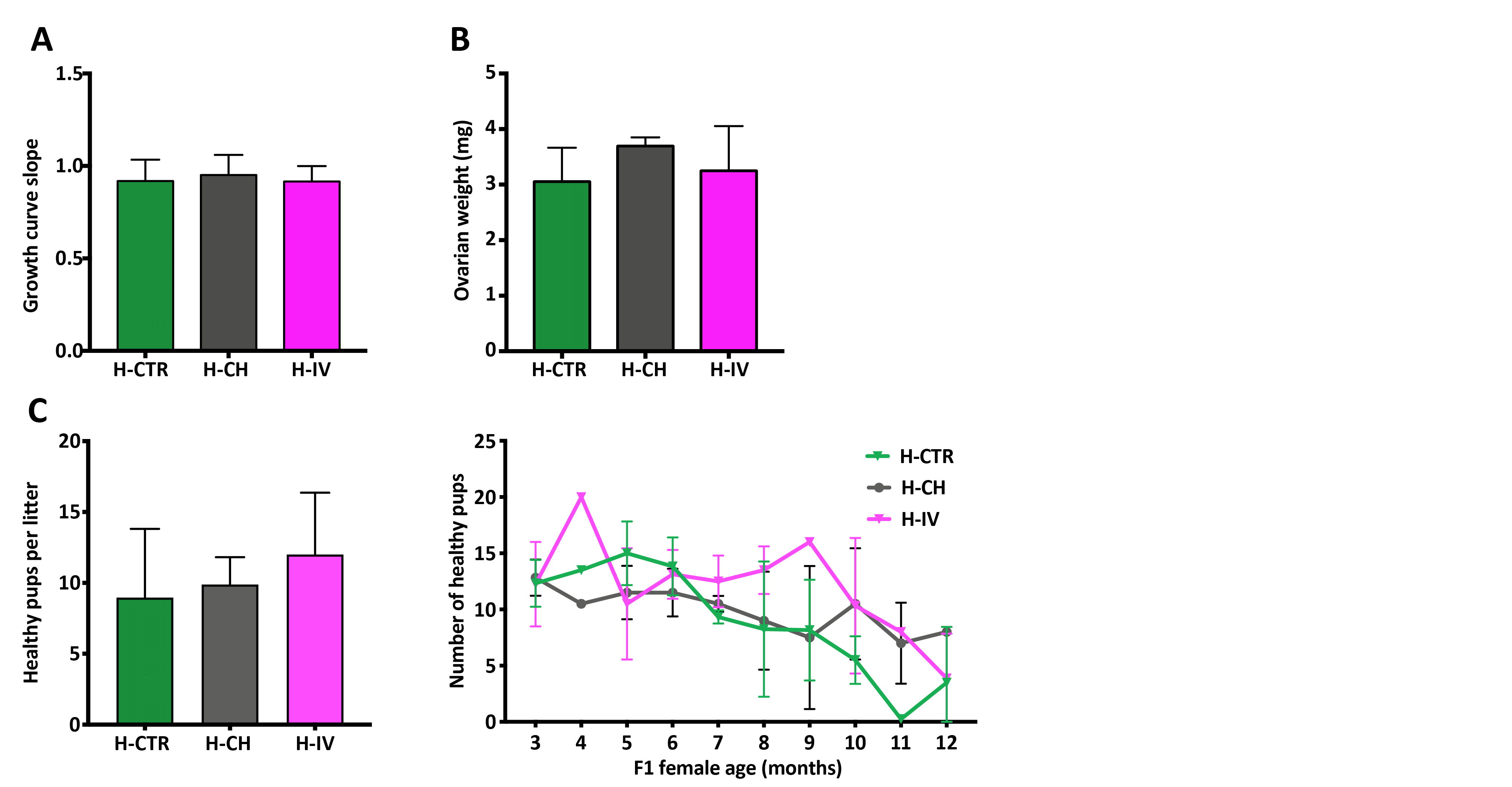

Supplement: Supplementary file 4 — Additional file 4: Figure S3. F1 study. A Slope of the growth curve of F1 females. Body weight was measured at day 21 and 35. Values are expressed as means±SD. Sample size: n≥3 where n is the number of litters analysed. B Weight of the ovaries from 35-day-old F1 females. Values are expressed as means±SD. Sample size: n≥3 where n is the number of litters analysed. C Number of F2 pups delivered from F1 females. Mean values for the whole period analysed are shown on the left panel, while the trend of the mean values overtime is shown on the right panel. Values are expressed as means±SD. H-CTR: n=3, H-CH: n=4, H-IV: n=4 where n is the number of F0 females analysed, for each of which data from two F1 females were averaged. [file 13287_2021_2590_MOESM4_ESM.png]
